# Looking Like a Leader–Facial Shape Predicts Perceived Height and Leadership Ability

**DOI:** 10.1371/journal.pone.0080957

**Published:** 2013-12-04

**Authors:** Daniel E. Re, David W. Hunter, Vinet Coetzee, Bernard P. Tiddeman, Dengke Xiao, Lisa M. DeBruine, Benedict C. Jones, David I. Perrett

**Affiliations:** 1 School of Psychology and Neuroscience, University of St Andrews, St Andrews, United Kingdom; 2 Department of Genetics, University of Pretoria, Pretoria, South Africa; 3 Department of Computer Science, University of Aberystwyth, Aberystwyth, United Kingdom; 4 Institute of Neuroscience and Psychology, University of Glasgow, Glasgow, United Kingdom; Northwestern University, United States of America

## Abstract

Judgments of leadership ability from face images predict the outcomes of actual political elections and are correlated with leadership success in the corporate world. The specific facial cues that people use to judge leadership remain unclear, however. Physical height is also associated with political and organizational success, raising the possibility that facial cues of height contribute to leadership perceptions. Consequently, we assessed whether cues to height exist in the face and, if so, whether they are associated with perception of leadership ability. We found that facial cues to perceived height had a strong relationship with perceived leadership ability. Furthermore, when allowed to manually manipulate faces, participants increased facial cues associated with perceived height in order to maximize leadership perception. A morphometric analysis of face shape revealed that structural facial masculinity was not responsible for the relationship between perceived height and perceived leadership ability. Given the prominence of facial appearance in making social judgments, facial cues to perceived height may have a significant influence on leadership selection.

## Introduction

Split-second judgments of competence from facial images are positively correlated with real-life electoral success [1]. Judgments of competence from briefly presented (i.e., 1/10 s) face images have predicted outcomes in elections for United States (US) congress [2], governor [3], and president [4]. Quick leadership judgments from faces have also been found to predict voting decisions in the United Kingdom [5], [6], Canada [7], Australia [8], Ireland [9], Italy [10], and Japan [11]. Children’s judgments of leadership can predict electoral success as well, and closely match leadership judgments made by adults [12].

Perception of leadership ability from facial images also correlates with leader success in the corporate world. Profits earned are regarded as a good indication of a business leaders’ ability [13]. Judgments of power from face images of business CEOs have been found to correlate with company profits in top American businesses [14], and similar judgments from faces of Managing Partners correlate with profits earned in law firms [15]. This relationship between facial appearance and leadership ability holds for both male and female faces [14], [16]. The relationship also exists even if facial images are taken years before a person gains their leadership position, suggesting that face characteristics that influence leadership selection are consistent across time and not developed during leadership roles [17]. Taken together, these findings suggest that facial appearance not only influences leadership selection in the political realm, but also predicts actual leadership ability in a corporate context.

Another physical characteristic related to leadership selection is body height. For example, taller candidates are more likely to gain a higher percentage of the popular vote in US presidential elections [18], and the difference in candidates’ heights predicted the difference in obtained presidential election votes from 1824 to 1992 [19]. Outside of politics, height predicts career success and income [20], [21]. Taller men and women run for positions of leadership more frequently [22], and are more likely to be selected to leadership positions within the business world [20]. Taller men and women are also more dominant and assertive [23] and less anxious [21]. Recent research has demonstrated that tall stature is correlated with higher perceived dominance, health and intelligence in men and higher perceived intelligence in women [24]. The association between height and dominance is present even in preverbal infants, who show more surprise when taller lines back away from shorter lines than vice versa in simulated confrontations [25]. The association between height and perceived leadership ability may reflect the correlation between physical size and rank in leadership hierarchies present throughout human history [22] and in several current primate species [26]–[29].

Given the relationship between leadership perceptions and both facial appearance and physical height, it is possible that facial cues to height play a role in leadership selection, especially in circumstances where bodies are occluded from view. Such situations are common; political candidates often stand behind podiums during speeches, sit at tables during debates and are often presented from the neck up on television and in campaign adverts. If cues to height are visible in faces, they may affect perceived leadership ability and bias leadership selection with anatomical information irrelevant to political acumen. Recent research has indicated that height can be assessed from face images [30]. The current study will examine the extent to which specific facial cues to perceived height (cues that influence how tall an individual appears from facial images) influence perceived leadership ability. Previous studies have found that perceived height from face images is correlated with perceived leadership ability in three-dimensional faces [31], while another study found that preferences for facial cues to taller perceived height are preferred in leaders’ faces more in war than peace contexts [32].

While judgments of height and leadership ability may be reliably drawn from faces, few studies have examined the specific quantitative face dimensions that influence these attributions. One possible facial cue that could be associated with height is facial elongation (the full length of the face divided by the width). Facial elongation increases from infancy to adulthood, as the lower jaw develops and protrudes from the face [33], [34] and faces become less round and more oval [35]. Facial elongation could therefore be a cue to height. We will examine if facial elongation influences perceived height and test the indirect impact this has on perceived leadership ability.

To investigate how height cues in the face influence leadership selection, one must control for other facial cues already known or suspected to influence perceived leadership ability. For example, recent research has demonstrated that facial width-to-height ratio (bizygomatic face width, with length of the face defined as the distance from the upper eyelid to the top of the upper lip, see Fig. S1.) predicts leadership success in businesses with low levels of management complexity [36] and predicts achievement drive in U.S. presidents [37]. Recent studies have found that business leaders in the United Kingdom have higher width-to-height ratios than age and sex-matched counterparts [38]. Facial width-to-height ratio correlates with perception of dominance [38] and aggressive and untrustworthy behaviour [39]–[41], traits that likely impact leadership success. It is therefore appropriate to consider the influence of facial width-to-height ratio when examining how facial cues to perceived height influence perceived leadership ability.

Another perceptual trait linked with leadership selection is facial maturity [14], [15], [17]. Baby-faced individuals appear less competent [42], [43], which could influence leadership perception [15], [42]. While previous studies have found that baby-faced individuals do not face disadvantages in actual leadership races [43], it may be useful to consider the role of facial maturity when assessing face traits that influence leadership judgments. We will therefore also control for facial maturity when assessing how facial cues to perceived height influence perceived leadership ability.

Finally, leadership selection is also influenced by perceived facial masculinity (sexual dimorphism in face shape). For example, masculine face structure is preferred in leaders’ faces in times of intergroup conflict, while more feminine faces are preferred during periods where within-group relationship maintenance is emphasized [6], [44], [45]. It is possible that cues to height are morphologically related to cues associated with masculinity, since men are, on average, taller than women in every culture studied to date [46], [47]. The current study will therefore assess whether facial cues to perceived height are morphologically distinct from those to facial masculinity.

## Study 1: Evaluating Height and Leadership Ability from Faces

In Study 1, we assessed if height can be perceived from facial cues alone, and if so, whether facial cues to perceived height also influence perceived leadership ability. First, men’s and women’s faces were rated for height and leadership ability. We then assessed whether these ratings were related to the actual height of the individuals photographed. We also assessed whether the actual age or sex of the person photographed and perceived facial maturity related to perceived leadership ability. Finally, we computed facial elongation and facial width-to-height ratio for each face presented to assess whether any of these dimensions predicted perceived height or leadership ability.

### Methods

All studies were cleared by the University of St Andrews ethics Committee. All participants provided written informed consent. All consent and debrief procedures were approved by the ethcis committee.

#### Stimuli

We presented participants with Caucasian face images of 47 men (mean age = 25.25 years, SD = 4.64 years, mean body mass index (BMI) = 24.10 kg/m^2^, SD = 3.52 kg/m^2^, 4 with partial beard) and 83 women (mean age = 23.04 years, SD = 3.81 years, mean BMI = 20.05 kg/m^2^, SD = 4.12 kg/m^2^) that were obtained from a commercially available database of face images (available at www.3d.sk). All individuals photographed had their hair pulled back and were photographed under constant lighting and camera set-up. Face images were standardized for inter-pupillary distance and cropped slightly below the chin. Men’s heights ranged from 168 cm to 192 cm (mean = 179.72 cm, SD = 6.43 cm), and women’s heights ranged from 156 cm to 184 cm (mean = 167.45 cm, SD = 6.33 cm).

#### Participants

Twenty-two Caucasian participants (11 men, 11 women, mean age = 25.32 years, SD = 2.47 years) were recruited from the School of Psychology at the University of St Andrews to rate the faces for height and leadership ability. All participants gave informed consent. Ten participants (5 men, 5 women, mean age = 24.07, SD = 1.70) independently rated the faces for maturity.

#### Procedure

Participants were presented with the 47 men’s faces and 83 women’s faces individually in two separate blocks. In one block, participants were asked to “Please rate how tall you think this person is in either feet and inches or cm” and were given eight evenly spaced height divisions from 152 cm to 203 cm (5′0″–6′8″). In another block, participants were asked to rate on a 1 (low) to 7 (high) Likert scale “how good of a leader do you think this person is?” Presentation order of the blocks was counterbalanced. Ten independent participants were asked to rate “How mature-looking is this person?” on a scale from 1 (extremely baby-faced) to 7 (extremely mature-faced). The trial order was randomized in the height, leadership and facial maturity blocks.

#### Face measurement

Facial elongation was defined as the full length of the face divided by the full width, and was measured for each face (Fig. S1). Face length and width were calculated using custom face-processing software [48]. We measured face length by calculating the maximum vertical distance between three x-y coordinates at the top of the forehead and the base of the chin. The width of the face was defined as the maximum horizontal distance between five coordinates outlining the perimeter of the left and right sides of the face (Fig. S1).

Face width-to-height ratio was measured in the same manner as previous papers [36], [39], [41], [49]. Facial width-to-height ratio was defined as the maximum vertical distance between the crease of the upper eyelid and the top of the upper lip divided by the maximum horizontal width across the sides of the face (Fig. S1). Facial width-to-height ratio was measured using the same face-processing software as facial elongation [48].

### Analysis

**Figure 1:**
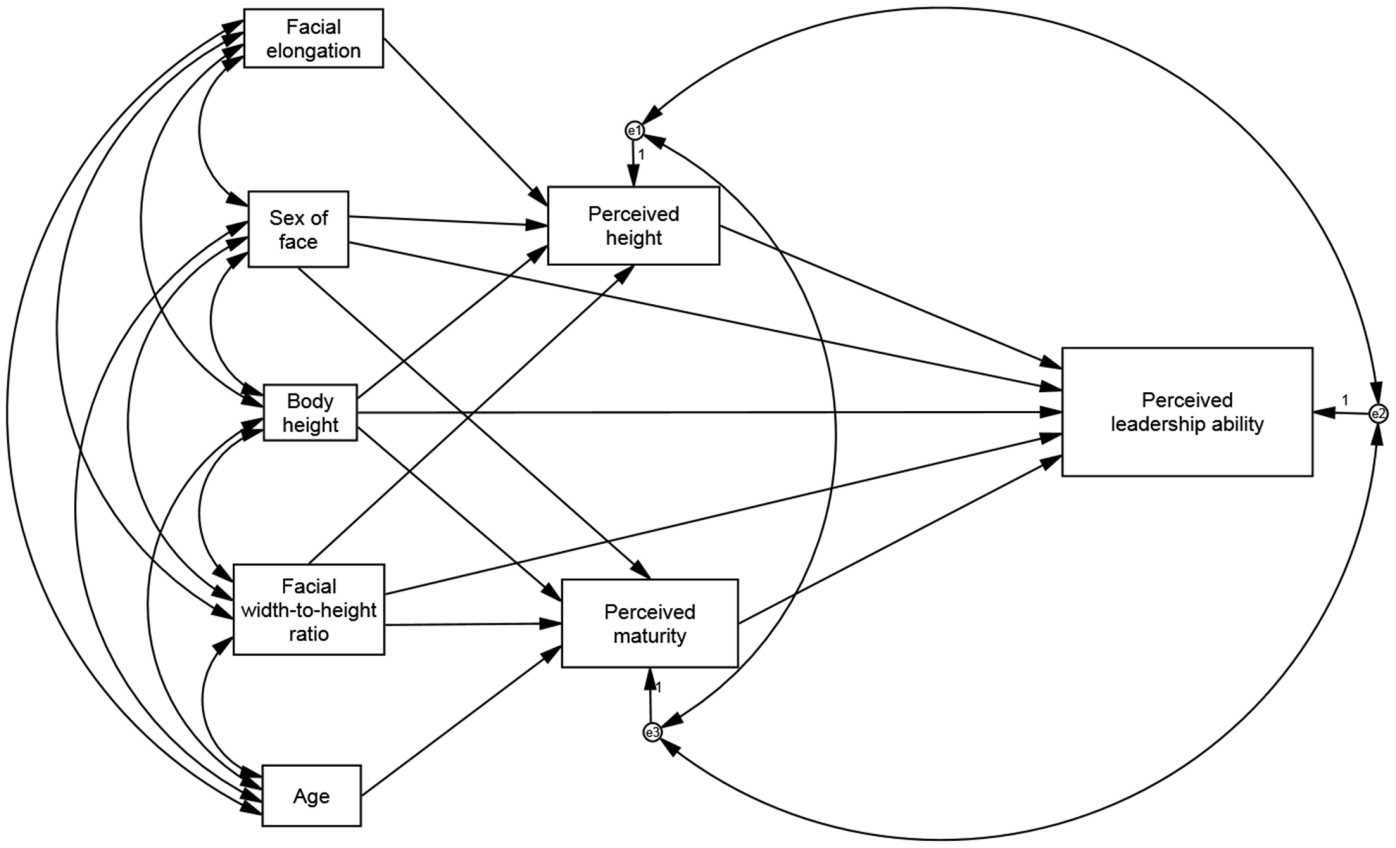
Path diagram outlining analyzed relationships between variables. Sex of face, facial width-to-height ratio, and body height, were examined for direct and indirect effects on perceived leadership ability. Endogenous variables (perceived height and perceived facial maturity) were examined for their direct effect on perceived leadership ability. Facial elongation and age were examined for their direct effects on perceived height and facial maturity, respectively.

Inter-rater reliability values were estimated for height, leadership and maturity ratings for each sex of face. A full mediational path analysis scale was constructed to assess direct and indirect effects of the variables on leadership judgments (Fig. 1). Sex of face, body height, facial elongation, facial width-to-height ratio and age were entered as exogenous variables. Given that perceived height and perceived maturity could be influenced by the other variables, they were entered as endogenous variables in a path analysis with structural equation modelling software (SPSS AMOS). We examined whether each of the following variables directly affected perceived leadership ability: perceived height, perceived facial maturity, sex of face, body height, and facial width-to-height ratio. We also examined whether sex of face, body height and facial width-to-height ratio had direct effects on perceived height and perceived maturity. The model assessed whether perceived height was influenced by facial elongation and whether perceived maturity was affected by age. Relationships with the weakest theoretical bases (elongation as a predictor of maturity and age as a predictor of perceived height) were omitted, as the mathematical constraints of the path diagram demanded the model not be saturated. Disturbance terms were entered for perceived leadership ability, perceived height, and perceived facial maturity, and the covariance between all disturbance terms was calculated.

### Results and Discussion

Inter-rater reliability was high for both perceived height ratings (n = 22, intraclass correlation coefficient (ICC) = 0.95) and perceived leadership ratings (n = 22, ICC = 0.97). Inter-rater reliability was also high for ratings of perceived facial maturity (n = 10, ICC = 0.88).

The path model (Fig. 1) fit the data well (χ^2^ = 1.29, p = 0.53, χ^2^/df = 0.64; Standardized RMR = 0.01; CFI = 1.00; RMSEA = 0.00, RMSEA 90% CI: 0.00–0.15).

The path analysis revealed that body height (β = 0.11, p = 0.02), sex of face (β = 0.74, p<0.01), and facial elongation (β = 0.19, p<0.01) all predicted perceived height, while facial width-to-height ratio did not (β = −0.04, p = 0.32). Age (β = 0.40, p<0.01), sex of face (0.37, p<0.01), body height (β = 0.20, p = 0.01) and facial width-to-height ratio (β = 0.17, p = 0.01) all had significant effects on perceived maturity.

Perceived height had a strong and significant effect on leadership ratings (β = 1.05, p = 0.04), while facial maturity ratings also correlated with perceived leadership ratings in the current sample (β = 0.48, p = 0.03). Of the exogenous variables, the sex of face showed a significant direct effect on leadership ratings, with men being perceived as better leaders than women (β = 0.93, p<0.03). Neither width-to-height ratio (β = −0.04, p = 0.73) nor body height (β = −0.15, p = 0.29) had significant direct effects on leadership ratings.

Since the exogenous variables could have influenced perceived leadership ability by affecting perceived height or maturity, we examined the indirect effects of exogenous variables on leadership ratings (Table 1). Impact of the indirect effects was classified by the criterion published by Stroud and Bolger [50]. The sex of face had a large indirect effect on leadership ratings (β = 0.95), as did body height (β = 0.21). Facial elongation had a moderate indirect effect on leadership ratings (via perceived height, β = 0.20), as did age (via perceived maturity, β = 0.19). Facial width-to-height ratio (β = 0.04) had a negligible indirect effects on leadership ratings. Disturbance terms between perceived height and facial maturity covaried at a significant level (β = 0.09, p<0.01), reflecting a positive correlation between perceived height and maturity. The disturbance terms for perceived height and perceived leadership did not show significant covariance (β = −0.03, p = 0.57); nor did the disturbance terms for perceived maturity and perceived leadership (β = −0.14, p = 0.09). See Table 1 for a full list of regression weights and significance values for all relationships between variables.

**Table 1:** Standardized regression estimates of the direct and indirect effects of exogenous variables on height, maturity and leadership ratings, and the direct effects of endogenous variables on leadership ratings.

Direct effects				Indirect effects
Exogenous variables	Perceived height	Perceived maturity	Perceived leadership ability	Perceived leadership ability
Body height	0.11*	0.20*	−0.15	0.21
Facial width-to-height ratio	−0.04	0.17**	−0.04	0.04
Sex of face	0.74**	0.37**	0.93**	0.95
Facial elongation	0.19**			0.20
Age		040**		0.19
**Endogenous variables**				
Perceived height	–	–	1.05*	–
Perceived maturity	–	–	0.48*	–

*p<0.05* = *, *p<0.01* = **.

Brand & Bradley [51] suggest that calculating relationships between ratings averaged across participants (such as the perceived height and leadership ratings in the path model) can inflate correlation estimates. To confirm any relationships, they suggest calculating the correlations between two variables of interest for each participant, then computing the average of these correlations. We therefore calculated the individual correlations between height ratings and leadership ratings for each participant, and then averaged those correlations together. Height and leadership ratings were significantly correlated within participants (n = 130, average r = 0.20, SEM = 0.03, t = 2.31, directional p = 0.01). The relationship between height and leadership held for both female and male participants (both average r≥0.15, both t≥1.72, both directional p<0.05). The relationship between height and leadership ratings was therefore significant at the participant level.

## Study 2: Manipulating Perceived Height to Maximize Perceived Leadership Ability

The faces in Study 1 were natural (i.e., unmanipulated) and not constrained to differences in shape. Skin colour and texture have a profound effect on facial judgments [52] and influence perceived health [53], [54], a trait that has been found to affect voting decisions when viewing avatars of biological motion [55]. We therefore assessed whether shape cues to perceived height affected perceived leadership ability when manipulated independently from surface information. In Study 2, we created synthetic faces and transformed them in shape only to manipulate their perceived height (as described below). First, we validated our perceived height transforms to ensure that they altered perceived height. We then allowed participants to manually manipulate perceived height in faces in order to maximize perceived leadership ability.

### Methods

#### Stimuli

The faces that were rated for height and leadership ability in Study 1 were delineated with 189 points using custom face-processing software [48]. Five male and five female face composites were created for transforming. Composites were created by averaging three male or three female faces together [56]. Face composites were used to avoid any experimental confounds that may be inherent in an individual face.

**Figure 2:**
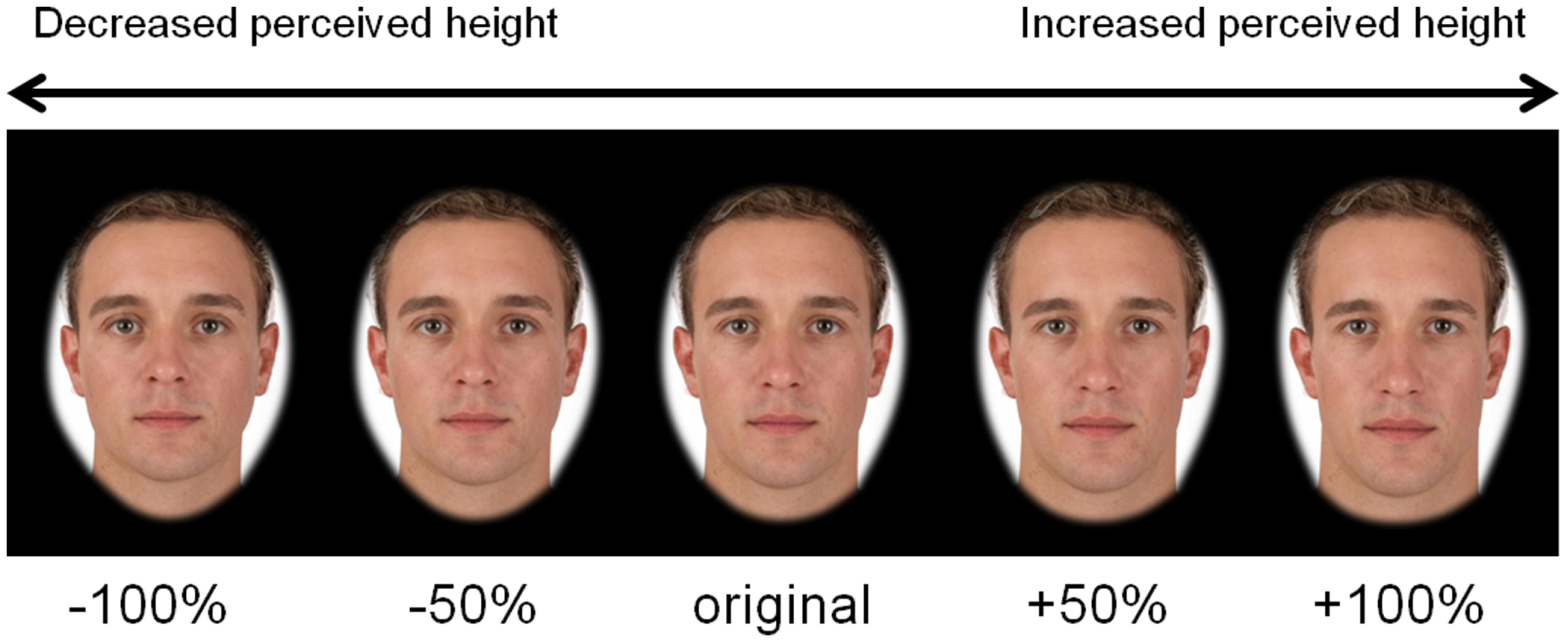
An abridged example of the perceived height transform used in Study 2. In the validation task, participants rated the heights of two male and two female composites transformed ±50% in perceived height. In the interactive task, participants were shown a composite and were asked to manipulate its shape to maximize perceived leadership ability. Transform levels of ±50% and ±100% are shown as examples, though participants could transform faces to any value between ±100% in the interactive task. On average, participants increased perceived height by 44.8% to maximize perceived leadership ability.

To create transforms, we averaged the faces of the 10 people who were perceived as shortest and the 10 people perceived as tallest within each sex (referred to as ‘perceived height prototypes’). Prototypes were matched for age and BMI. We created face shape continua of 20 steps for each of the 10 composites by applying ±100% of the shape difference between the perceived height prototypes of the same sex [56], [57]. This created face continua spanning from 100% ‘perceived short’ shape to 100% ‘perceived tall’ shape for each composite while maintaining the same identity (Fig. 2). The transforms manipulated faces in perceived height shape alone, leaving all other face parameters such as colour and texture constant. These techniques have been used successfully to manipulate perceived height in previous studies [57].

#### Manipulation check

A manipulation check was conducted to determine whether the height transform did in fact alter perceived height. In this, 16 women and 6 men (mean age: 28.91, SD: 10.96) participated in an online test to rate the height of faces transformed ±50% in perceived height. These participants were presented with individual images of two male and two female composites transformed ±50% in perceived height (Fig. 2). Participants were asked to rate how tall each person was on a scale of 1 (extremely short) to 7 (extremely tall). There was an average rating of 3.37 (SD = 0.73) for women’s composites decreased 50% in perceived height and 4.84 (SD = 1.01) women’s composites increased 50% in perceived height. There was an average rating of 4.06 (SD = 0.84) for men’s composites decreased 50% in perceived height and 5.06 (SD = 0.67) for men’s composites increased 50% in perceived height. Paired-samples t-tests revealed that the composites transformed to increase perceived height were rated as taller than those transformed to decrease perceived height for both women’s and men’s faces (both t(21)≥5.07, both p<0.01, both Cohen’s d≥2.21). Thus, our perceived height transforms did reliably alter perceived height, as in previous studies [55].

#### Participants

Twenty separate Caucasian participants (10 men, 10 women, mean age = 26.85 years, SD = 4.19 years) participated in an interactive leadership task. All participants gave informed consent.

#### Procedure

The participants completed an interactive task that required them to manually manipulate perceived height to maximize perceived leadership ability. A custom software program allowed participants to scroll over all 10 face composites (five men, five women) to view the 20 steps in that face’s continuum (Fig. 2), giving the perception that participants were manually transforming face shape. These transforms ensured that participants were only able to alter faces on one dimension (perceived height), while not changing skin colour or texture. We asked participants to transform each composite to make it “most like the person you would perceive as a good leader.” The initial face presented for each trial was randomized for starting degree of transformation. Scroll direction for transformation was also randomized so that scrolling the same way for each composite would not have the same transformation effect (for example, scrolling left may increase perceived height for one trial and decrease perceived height for the next trial). Participants were encouraged to view the whole transform continua before making a selection. The order of presentation of the 10 face composites (five male, five female) was randomized.

#### Analysis

Each composite had continua of 20 images spanning from 100% ‘perceived short’ shape to 100% ‘perceived tall’ shape. We calculated the average degree of transform used to maximize perceived leadership ability for each composite. One-sample t-tests were conducted to test how each composite was transformed against chance (no transformation).

### Results and Discussion

One-sample t-tests against chance (0% transformation) found that all ten composites were increased in perceived height to maximize perceived leadership ability (all t(19)≥3.00, all p<0.01, all Cohen’s d≥1.37). On average, faces were increased in perceived height by 44.8% (SD = 12.7%, range = 22.6% to 64.8%). A repeated-measures ANOVA showed no effects of the sex of the face (F(1, 18) = 2.90, p = 0.11, η_p_^2^ = 0.14) nor the sex of participant (F(1, 18) = 0.30, p = 0.59, η_p_^2^ = 0.02) on the degree of transform, and found no significant interaction between these factors (F(1, 18) = 1.31, p = 0.27, η_p_^2^ = 0.07).

Study 1 found that faces appearing to belong to taller people were rated as better leaders. In Study 2, participants altered face shape in a way that affected perceived height while retaining the same skin colour and texture information and keeping the identity of the face constant. Participants increased perceived height in all faces to maximize perceived leadership ability. Participants increased face shape associated with taller height by an average of nearly 45% to maximize leadership perception, confirming the relationship between perceived height and leadership ability in faces.

## Study 3: Morphological Face Cues to Height and Masculinity

Previous studies have demonstrated that facial cues to masculinity (or sexual dimorphism) affect perceived leadership ability [6], [44], [45]. We examined whether height and masculinity are morphologically distinct facial cues, and whether morphological masculinity is related to perceived height and leadership judgments. It is important to note that morphological differences between male and female faces may be distinct from cues that influence perception of masculinity. Indeed, recent studies have demonstrated that morphological masculinity scores do not predict perception of masculinity in male faces [58]. The purpose of Study 3 is to determine whether morphological face shape differences associated with physical height are equivalent to or distinct from face shape differences between men and women.

Determining morphological cues to masculinity has been achieved in other studies through principal component analysis (PCA) of face shape and canonical discriminant analysis (CDA) distinguishing the sex of face [59]. We followed similar methods to establish morphological masculinity “scores” for the faces that were rated for height and leadership ability. We then assessed whether masculinity scores were related to height, perceived height, or perceived leadership ability. Study 3 differs from the previous studies in that structural masculinity is computationally calculated, and masculinity scores are then compared across height and leadership ratings collected in Study 1.

### Methods

#### Morphological masculinity analysis

A morphometric analysis of facial masculinity was conducted on the faces rated for height and leadership ability. The morphometric analysis followed established methods [59]. Face delineations were reduced to 137 x-y coordinates (Fig. S1), eliminating more subjective points from the original delineations and keeping those outlining prominent structural facial features. The face delineations used here included 8 landmarks not used in other morphometric analyses [58], [59] that provide more resolution to the nose and face perimeter outlines. Each face was then aligned using Procrustes alignment to eliminate variances due to scale, translation and rotation. The face shapes were then parameterized using principal components analysis. Sixteen principal components (PCs) were selected using Kaiser-Guttman criteria. Each face is thus described as a set of parameters of the 16 retained PCs. Canonical discriminant analysis (CDA) was then performed on the retained PCs to distinguish male and female faces. Morphological masculinity “scores” were created from the CDA output. Masculinity scores were centred on 0, with scores below 0 indicating feminine face structure, and scores above 0 masculine face structure. The further a score deviated from 0, the more morphologically feminine (if below 0) or masculine (if above 0) the face was. See [59] for more details on producing morphological masculinity scores from face stimuli.

#### Analysis

Masculinity scores were assessed for predictive validity. On the recommendations of Brand & Bradley [51], we calculated the correlations between structural masculinity scores and the perceived height and leadership ratings for each participant in Study 1. Individual correlations were then averaged to determine the overall correlation between structural masculinity and perceived height or leadership ability. Since structural masculinity scores were defined by differences between women’s and men’s face shape, we calculated averaged correlations for women’s and men’s faces separately. We also calculated the correlation between structural masculinity and body height for women’s and men’s faces.

### Results and Discussion

Morphological masculinity scores were based on 16 principal components (PCs) explaining 89.0% of variance in face shape. Masculinity scores correctly predicted sex for 97.4% of faces.

Morphological masculinity scores did not correlate with leadership ratings in either women’s (n = 83, averaged r = 0.05, SEM = 0.02, p = 0.67) or men’s (n = 47, averaged r = −0.05, SEM = 0.02, p = 0.74) faces. Morphological masculinity scores did not correlate with perceived height ratings in either women’s (n = 83, averaged r = −0.09, SEM = 0.02, p = 0.42) or men’s (n = 47, averaged r = −0.11, SEM = 0.03, p = 0.46) faces. Morphological masculinity was not correlated with body height for women’s (n = 83, r = 0.11, p = 0.35) or men’s (n = 47, r = −0.20, p = 0.19) faces.

**Figure 3:**
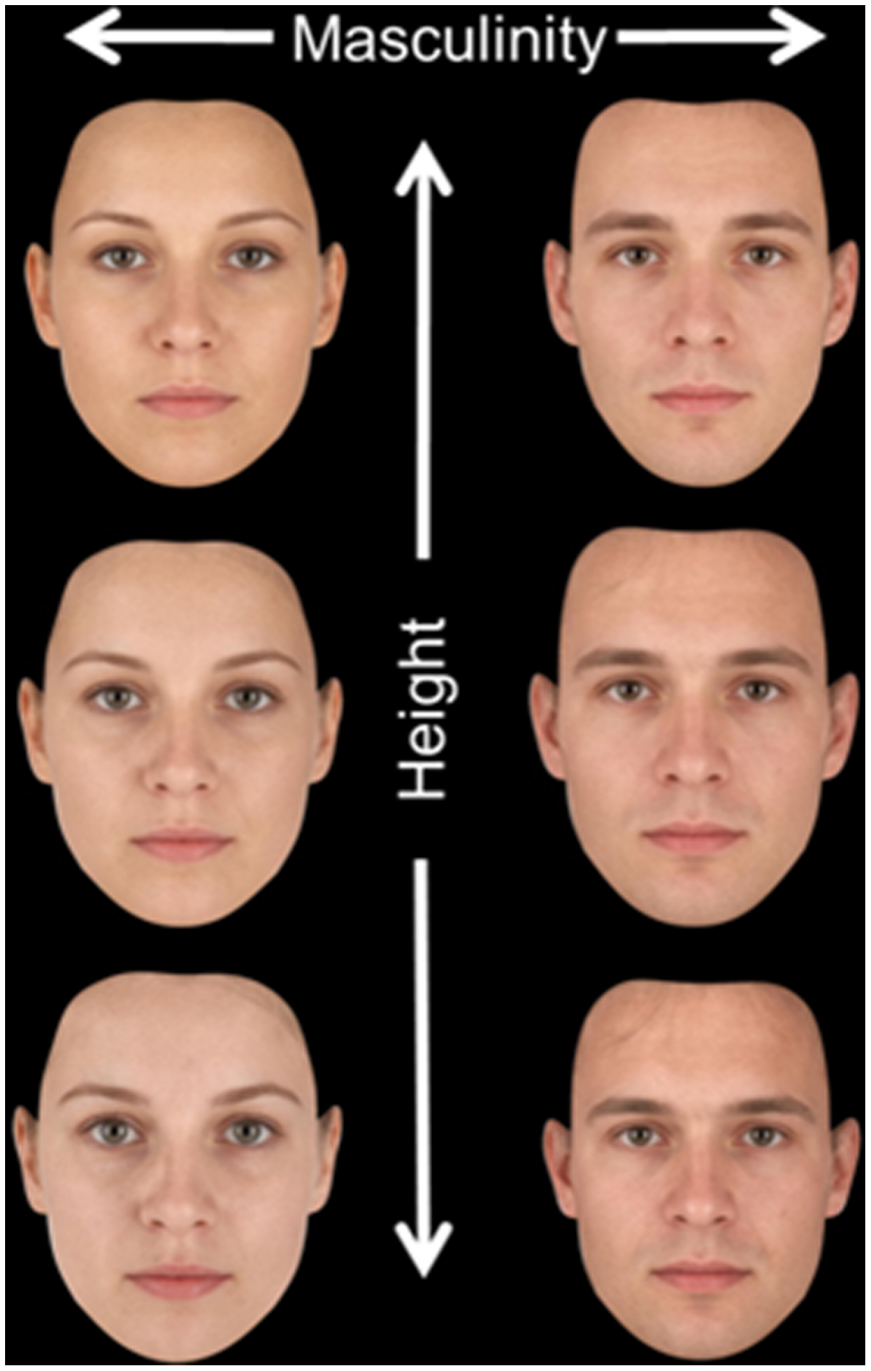
An averaged female and male face (middle row), and averaged faces of the 10 shortest (bottom row) and tallest (top row) individuals for each sex. Height and masculinity are morphologically distinct parameters in the face.

Morphological masculinity scores correctly predicted sex of face but did not differ with actual height. Masculinity scores showed no relationship with perceived height or leadership ratings. While morphological masculinity does not equate to perceived masculinity [58], Study 3 indicates that facial cues to perceived height are distinct from the morphological shape differences between male and female faces (Fig. 3). Thus, the link between perceived height and leadership perception cannot be due to morphological shape differences between male and female faces.

## General Discussion

Study 1 found body height, facial elongation, and sex of face all impact perceived height, and that perceived height has a strong effect on perceived leadership ability. Study 2 found that, when altered in isolation, structural facial cues that increase perceived height are enhanced to maximize perceived leadership ability. Study 3 revealed that the relationship between perceived height and perceived leadership ability cannot be accounted for by the morphological differences between men and women’s faces.

Perceived height from faces images had a very strong relationship with perceived leadership ability (β = 1.05) as found in previous studies [31], [32], and actual height did have a significant impact on perceived height, yet actual height had no other relationship with leadership ratings. Face cues are often overgeneralized for efficient processing at the cost of accurate inferences [60], [61]. It is possible that face cues to body height, such as facial elongation, are overgeneralized. Indeed, the relationship between facial elongation and perceived height is stronger than that between body height and perceived height. Such a phenomenon would explain how actual height can predict perceived height, yet lack a significant relationship with perceived leadership. These findings emphasize the possible importance of facial cues to height in democratic leadership selection. While leader candidates’ heights are often unknown or visually obscured in political forums (for example, electoral candidate debates now often take place at tables to offset visible height differences), their faces are often on display in various campaign advertisements and media appearances. Previous research indicates that facial appearance has a great impact on social judgments like attractiveness, maybe more so than body characteristics [62]–[64]. The current studies suggest facial cues to perceived height have a large effect on leadership ratings. Future work could discern the relative impact of facial cues to perceived height and actual body height in overall leader judgments.

While two previous studies have examined how facial cues to perceived height influence leadership selection [31], [32], this is the first study to quantify measurable face dimensions that influence height judgments. Faces grow longer and proportionately narrower throughout childhood while the body grows taller [33]–[35], which led to the current hypothesis that facial elongation would influence perceived height. The path analysis in Study 1 revealed this to be the case, as elongation had a strong effect on height judgments (β = 0.19). To the authors’ knowledge, facial elongation is the first quantifiable cue to the perception of height from faces reported in the literature.

The sex of the photographed individual had a significant direct effect on perceived leadership ability, with men being perceived as better leaders than women. Men’s faces are generally perceived to be more dominant than women’s faces [65], and dominance is correlated with perceived leadership ability in faces [1], [14]. Men are generally preferred as leaders when groups face an external threat such as war, likely because they are viewed as more dominant and aggressive [66]. While women are more likely to adopt a democratic leadership style [67] and are preferred as leaders when the maintenance of intragroup relations is emphasized [68], men are quicker to claim leadership roles, even when a woman seems more qualified [69]. Women still struggle to attain leadership roles, despite increasing numbers in the workforce [70], and men hold the majority of leadership positions around the world [71]. The current results suggest the bias towards male leadership extends to facial images.

Perceived facial maturity was found to correlate with leadership ratings. Facial maturity correlates with perceived competence [1], [42] and power [14], [15], [17], and increasing babyfacedness in politicians’ faces decreases perceived dominance and strength [72]. While some studies have found a relationship between facial maturity and voting behaviour [11], other studies have found that facial maturity does not predict electoral success [43]. The current study suggests that facial maturity predicts perceived leadership ability within a sample of young adults.

Facial width-to-height ratio was not related to perceived leadership ability in the current study. Facial width-to-height ratio is a correlate of actual leadership success [36], is higher in business leaders than the general age- and sex-matched populace [38], and predicts achievement drive in U.S. Presidents [37]. Facial width-to-height ratio also positively correlates with measures of aggressiveness and untrustworthy behavior [39]–[41]. Whereas facial width-to-height ratio correlates with actual measures of leader success and ambitious and aggressive behavior, the current study finds that it does not influence perceived leadership ability. Since facial width-to-height ratio correlates with aggressiveness, it is possible people with high width-to-height ratio would be perceived as good leaders if a situation in which aggressive leadership (i.e.- a war context) is called for. Future research could elucidate how facial width-to-height ratio impacts perceived leadership ability under different leadership contexts.

Study 3 demonstrated that structural masculinity, as measured by morphometric analysis, was not responsible for the relationship between perceived height and perceived leadership ability. It is important to note that measures of structural masculinity in faces do not necessarily equate to perceived masculinity [58]. It is therefore possible that facial cues to perceived height correlate with those to perceived masculinity, which has been linked to leadership ratings in previous studies [6]. Recent research has demonstrated that facial cues to perceived height and masculinity have non-equivalent effects on perception of dominance [32], suggesting these two perceptual traits are distinct. Future research could elucidate the relationship between perceived height and perceived masculinity, and investigate their relative impacts on perceived leadership ability.

The current studies show a strong positive relationship between perceived height and leadership ability; however it is important to note some limitations. While attempts were made to control for possible face parameters that could influence perceived leadership ability in Study 1, it would be impractical to assess faces for all possible variables. For example, facial attractiveness influences perceived competence, which impacts leadership selection [1]. Facial attractiveness influences leadership selection differently under different social contexts, as people choose leaders with attractive faces more in a war context than a peace context [73]. Ratings of facial attractiveness have correlated with election votes obtained in Australia [74], Finland [75], and the United Kingdom [5]. Perceived height has been found to influence facial attractiveness, with both women and men preferring male faces altered to increase perceived height [57]. However, while Re et al. [57] found that participants increased perceived height by 15.12%–21.15% to maximize attractiveness, Study 2 used the same transforms and found that participants altered face shape to increase perceived height by an average of 44.8% to maximize perceived leadership ability using similar transforms. Furthermore, Re et al. [57] found that participants reduced perceived height to maximize attractiveness in women’s faces, while perceived height was increased to maximize perceived leadership ability in both women’s and men’s faces. These studies suggest that while manipulating perceived height affects facial attractiveness, attractiveness cannot explain the relationship between perceived height and perceived leadership ability.

It is important to note that the current study lacked ratings of dominance and competence. Leadership judgments from face images are likely altered by the appearance of dominance and competence [1], [76]. Indeed, several studies have found that impressions of dominance and power correlate with real-world leadership success [14]–[17]. The relationship between physical height and leadership rank is also likely due to impressions of dominance [22], as taller people self-report more dominant and assertive behavior [23]. Previous research has demonstrated that increasing cues to perceived height increases perceived dominance [32]. We speculate that the relationship between perceived height and leadership ability may be mediated by dominance (i.e.-making someone look taller also makes them look more dominant, and thus more leader-like). Indeed, the influence of perceived facial masculinity and maturity on leadership selection is likely also due to associations with dominance and power [6], [14], [44], [45]. Perception of dominance is undoubtedly related to leadership selection, and further research could elucidate the nature of the relationship between leadership choice, perceived dominance, and judgments of height, maturity, and masculinity.

Previous research has found that social judgments drawn from facial features can impact leadership choice. For example, judgments of trustworthiness and warmth influence perceived leadership ability [11], [16], [73], and emotional expression affects these impressions [77]. Furthermore, facial features that enhance perceived competence (eyes closer to eyebrows, higher cheekbones, angular jaws) also likely impact leadership judgments [1]. The face stimuli used here were all holding neutral (non-emotive) expressions, and Study 2 controlled for emotional variance while altering face shape. While the current study focused primarily on face shape cues to perceived height and leadership ability, the impact of emotional expression and social judgments drawn from the internal facial features cannot be overlooked when examining the effect of faces on leadership selection.

The current study did not specify the context of leadership selection. Previous studies find that facial characteristics are differentially favoured in leaders’ faces in varying social contexts. For example, people choose leaders with higher facial masculinity and facial attractiveness in an intergroup conflict context such as war, but choose leaders with more feminine and trustworthy faces in a peace context or when intragroup conflict must be resolved [6], [44], [45], [73]. In accordance with this, one previous study found that preferences for tall-looking leaders was greater in a war context [32]. The current research demonstrates that the relationship between perceived height and leadership exists outside a specific leadership context.

Facial appearance has a strong effect on leadership selection. Judgments of leadership from face stimuli have been found to predict real political election outcomes from around the world (see [1], and predict leadership success in the corporate world [14]–[16]. Physical body height also correlates with leadership rank in politics and business [20], [22], [78]. The current study finds that face shape cues that make an individual appear taller also make them appear to be a better leader. It is therefore conceivable that individuals with facial characteristics associated with tall physical height are more likely to be selected as leaders in real political and corporate contexts. Furthermore, given the relationship between the appearance of leadership and real-world leadership success [14], [15], it is possible that facial cues to perceived height correlate with actual leadership acumen. While the relative impact of body height and face cues to perceived height on leadership selection is a subject of further research, faces generally have large effects on social judgments, perhaps more so than body stimuli [63]. Given the relative prominence of faces, and given that visible body height is often obscured, the current results suggest that facial cues to perceived height could have a great effect on real-world leadership selection.

## Supporting Information

Figure S1 **An example of face length and width measurements.** Face elongation was defined as the maximum vertical distance between three coordinates on the forehead and three coordinates on the chin, divided by the maximum horizontal distance between five coordinates alongside the perimeter of the face on the left and right sides (red lines). Facial width-to-height ratio was defined by the maximum vertical distance between the upper lip and upper eyelid (blue line) divided by the maximum width. The 137 delineation points used in the morphometric masculinity analysis are also shown.
(TIF)
