# Supplementary figures and images for: Looking Like a Leader–Facial Shape Predicts Perceived Height and Leadership Ability
Source: PLoS One. 2013 Dec 4;8(12):e80957. doi: 10.1371/journal.pone.0080957 (PMC3851990; doi:10.1371/journal.pone.0080957)

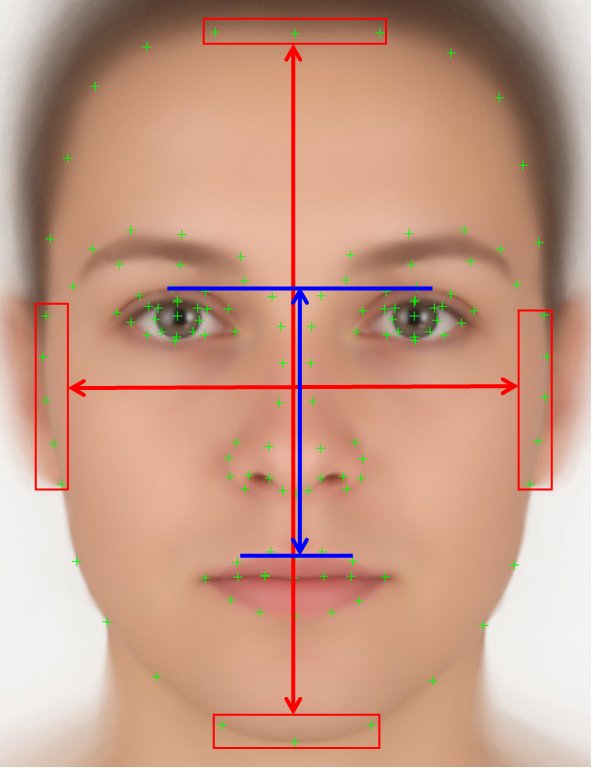

Supplement: Figure S1 — An example of face length and width measurements. Face elongation was defined as the maximum vertical distance between three coordinates on the forehead and three coordinates on the chin, divided by the maximum horizontal distance between five coordinates alongside the perimeter of the face on the left and right sides (red lines). Facial width-to-height ratio was defined by the maximum vertical distance between the upper lip and upper eyelid (blue line) divided by the maximum width. The 137 delineation points used in the morphometric masculinity analysis are also shown. (TIF) [file pone.0080957.s001.tif]
